# The utility of PSA density for selection of targeted versus systematic transperineal prostate biopsy: A retrospective cohort study

**DOI:** 10.1002/bco2.70247

**Published:** 2026-07-14

**Authors:** Johnny Wang, Narmina Khanmammadova, Karim Hanna, Ashley Gao, Angelina Wang, Kimberly Tran, Mohamad Ibrahim, Mackinnly Knoerzer, Kristene Myklak, Sohrab Ali, Michael Daneshvar, Mohammed Shahait, David Lee

**Affiliations:** ^1^ Department of Urology University of California Irvine Orange California USA; ^2^ Washington University School of Medicine St. Louis Missouri USA; ^3^ Golden State Urology Fremont California USA

**Keywords:** magnetic resonance imaging, prostate biopsy, prostate cancer

## Abstract

**Objectives:**

The objective of this study is to evaluate PSA density (PSAD) as an adjunctive predictor of prostate cancer (PCa) detection on magnetic resonance imaging (MRI)‐guided transperineal biopsy and to identify PSAD thresholds across which systematic sampling may be safely omitted.

**Methods:**

Data were collected from men who underwent MRI‐ultrasound software‐assisted fusion transperineal biopsy from July 2023 to August 2025. The cancer detection rate of targeted, systematic and combined strategies was stratified by PSAD. Multivariable logistic regression was used to identify predictors of grade group (GG) ≥ 2 and GG ≥ 3 PCa.

**Results:**

Among 379 men undergoing biopsy, overall GG ≥ 2 detection increased with PSAD: 29.2% (<0.1), 42.7% (0.1–0.1499), 61.2% (0.15–0.1999) and 78.4% (≥0.2). On multivariable analysis, PSAD was associated with higher odds of GG ≥ 2 (OR 2.4, 95% CI 1.73–3.3, *p* < 0.001) and GG ≥ 3 (OR 2.3, 95% CI 1.65–3.2, *p* < 0.001) PCa. At PSAD ≥ 0.2, the inclusion of systematic biopsy increased detection of GG 1 cancer by 42.3% but only increased detection of GG ≥ 2 and GG ≥ 3 cancer by 7.2% and 6.2%, respectively. Among 96 men who underwent radical prostatectomy, combined biopsy demonstrated the strongest grade group concordance at prostatectomy (κ = 0.747), though the difference between targeted and combined biopsy narrowed among those with PSAD ≥ 0.2 (κ = 0.628 vs. 0.584).

**Conclusion:**

PSAD is an accessible metric that may guide risk stratification and biopsy strategy selection. A PSAD threshold of 0.2 may identify those in whom systematic sampling increases detection of low‐grade disease with limited incremental yield for clinically significant disease.

## 1 INTRODUCTION

The use of magnetic resonance imaging (MRI)‐targeted prostate biopsy has significantly improved detection of clinically significant prostate cancer (PCa).^1^ Current guidelines recommend the combination of targeted and systematic biopsies for optimal detection of clinically significant disease.^2^, ^3^ However, routine inclusion of systematic cores may not only increase diagnosis of indolent disease but also expose patients to additional discomfort and morbidity.^4^, ^5^, ^6^, ^7^

Recent efforts have sought to identify men in whom systematic sampling may be safely omitted.^8^, ^9^ There remain limitations with reliance on MRI alone, as patients with Prostate Imaging Reporting and Data System (PI‐RADS) 3 lesions retain a significant likelihood of harbouring higher grade cancer.^10^, ^11^

PSA density (PSAD) is a robust marker of PCa that is shown to enhance risk stratification in conjunction with MRI.^12^, ^13^ Some data suggest that PSAD can identify patients in whom systematic sampling could be reduced without sacrificing detection of aggressive disease.^14^, ^15^ This strategy has not been validated for transperineal biopsy, which is increasingly preferred over the transrectal approach given comparable cancer detection, lower procedural morbidity and potential for greater cost‐effectiveness.^16^, ^17^ Landmark studies supporting the use of transperineal biopsy like the PREVENT trial have not reported on the differences in cancer detection rate for targeted and systematic biopsy or stratified by risk markers like PSAD.^16^ We hypothesize that PSAD may identify patients who can safely forgo systematic sampling.

## 2 PATIENTS AND METHODS

### 2.1 Study cohort

After Institutional Review Board approval (Protocol No. 3398), we retrospectively reviewed an institutional database of men who underwent MRI–ultrasound fusion transperineal prostate biopsy at a tertiary academic centre between July 2023 and August 2025 for clinical suspicion of PCa. Patients were excluded sequentially if they received targeted‐ or systematic‐only biopsy (*n* = 44), harboured no MRI‐visible lesions (*n* = 5) or underwent biopsy as part of an active surveillance protocol (*n* = 58).

### 2.2 MRI protocol

Imaging studies were obtained within our institution or from outside imaging centres prior to referral. When performed at our institution, MRI was performed using a 3.0‐T system without endorectal coil, with sequences including triplanar T2‐weighted, dynamic contrast–enhanced and diffusion‐weighted imaging. Outside studies were reviewed in their original format without protocol harmonization. All examinations were interpreted using PI‐RADS Version 2.0 or 2.1, and suspicious lesions were assigned a score of 1 to 5.

### 2.3 Biopsy protocol

All patients had at least one MRI‐visible lesion (PI‐RADS score ≥ 3). Prostate segmentation and annotation of regions of interest (ROI) were performed by the interpreting radiologist or urologist. Transperineal biopsies were performed using the Koelis Trinity® software fusion biopsy platform (Koelis, Meylan, France) under local or general anaesthesia. Targeted cores were taken first from the ROIs, followed by 10–12 systematic cores modelled after an extended sextant template (Figure S1). The number of targeted cores was individualized based on urologist discretion. Biopsy specimens were reviewed by dedicated genitourinary pathologists and reported using standard‐of‐care nomenclature. Results of targeted and systematic biopsies were separately recorded and coded on the one to five International Society of Urological Pathology (ISUP) GG scale.

### 2.4 Definitions and covariates

Covariates included age, race, family history of PCa, PSA, prostate volume and index PI‐RADS score. The index lesion was defined as the lesion with the highest PI‐RADS score; if multiple lesions shared the highest score, the lesion with the greatest maximal diameter was selected. Prostate volume was calculated from three‐dimensional MRI measurements using the ellipsoid formula. Clinically significant prostate cancer (csPCa) refers to GG ≥ 2 PCa.

### 2.5 Statistical analysis

Differences in characteristics were compared across patients with or without csPCa using the independent samples *t* test, Mann–Whitney *U* test or chi‐squared test. Multivariable logistic regression was used to evaluate the relationship between PSAD and GG ≥ 2 and GG ≥ 3 PCa. PSAD was log‐transformed with a base of 2 to fit a logistic regression curve and improve interpretability of the models. PSA was excluded from multivariable analysis to avoid collinearity with PSAD. In addition to risk stratification, we assessed PSAD as a tool for biopsy template selection by comparing cancer detection rates across PSAD strata. Prior to analysis, we defined a clinically acceptable risk–benefit profile for omitting systematic biopsy as <10% risk of missing GG ≥ 2 disease and a >30% reduction in detecting GG 1 disease, based on National Comprehensive Cancer Network guidance for PCa biomarker validation.^3^ A subgroup analysis was also performed for patients who underwent radical prostatectomy to assess concordance between the highest GG identified with each biopsy strategy and the final histopathology. The concordance outcome was defined as an exact GG match and was assessed using quadratic weighted Cohen's kappa, which accounts for the ordinal nature of GG classification by penalizing larger grade discrepancies more heavily. All analyses were performed per‐patient. Analyses were performed using R Version 4.4.0 (R Foundation for Statistical Computing, Vienna, Austria) and IBM SPSS Statistics for Windows, Version 29.0 (IBM Corp., Armonk, NY).

## 3 RESULTS

### 3.1 Cohort characteristics

In total, 379 patients with PI‐RADS ≥3 lesions underwent combined biopsy. Most of the cohort was biopsy‐naïve (*n* = 334/379, 88.1%), with the remainder having a history of prior negative biopsy (*n* = 45/379, 11.9%). Clinical characteristics of patients with and without csPCa are compared in Table 1. Prior to biopsy, patients with csPCa had greater PSA (median 6.7 ng/ml [IQR 5–11.5] vs. 5.5 ng/ml [IQR 4.1–7.9], *p* < 0.001), lower prostate volume (42 cc [32–63] vs. 56.3 cc [39–83], *p* < 0.001) and greater PSAD (0.16 ng/ml/cc [0.1–0.25] vs. 0.09 ng/ml/cc [0.07–0.14], *p* < 0.001).

**TABLE 1:** Baseline characteristics.

Variable	No csPCa (*N* = 192)	csPCa (*N* = 187)	*p* value
Age, years, mean (SD)	66 (7.6)	70 (8.0)	**<0.001**
Race, *n* (%)			0.17
White	114 (59.4%)	128 (68.4%)	
Black	4 (2.1%)	4 (2.1%)	
Asian/Pacific Islander	39 (20.3%)	35 (18.7%)	
Other/unknown	35 (18.2%)	20 (10.7%)	
Ethnicity, *n* (%)			0.076
Hispanic/Latino	27 (14.1%)	17 (9.1%)	
Not Hispanic/Latino	155 (80.7%)	166 (88.8%)	
Unknown	10 (5.2%)	4 (2.1%)	
Family history of prostate cancer, *n* (%)	43 (22.4%)	40 (21.4%)	0.8
Biopsy history			**<0.001**
Biopsy‐naïve	158 (82.3%)	176 (94.1%)	
Prior negative	34 (17.7%)	11 (5.9%)	
PSA, ng/ml, median (IQR)	5.5 (4.1–7.9)	6.7 (5–11.5)	**<0.001**
MRI prostate volume, ml, median (IQR)	56.3 (39–83)	42 (32–63)	**<0.001**
PSA density, ng/ml/cc, median (IQR)	0.09 (0.07–0.14)	0.16 (0.1–0.25)	**<0.001**
Index PI‐RADS score, *n* (%)			**<0.001**
PI‐RADS 3	66 (34.4%)	17 (9.1%)	
PI‐RADS 4	100 (52.1%)	85 (45.5%)	
PI‐RADS 5	26 (13.5%)	85 (45.5%)	
PSA density interval, *n* (%)			**<0.001**
	101 (52.6%)	43 (23.0%)	
0.1–0.1499	52 (27.1%)	37 (19.8%)	
0.15–0.1999	19 (9.9%)	30 (16.0%)	
≥0.2	20 (10.4%)	77 (41.2%)	
Highest biopsy grade group, *n* (%)			–
Benign	119 (62%)	–	
1	73 (38%)	–	
2	–	82 (43.9%)	
3–5	–	105 (56.1%)	
Targeted biopsies, median (IQR)	4 (4–6)	5 (4–8)	**0.003**
Positive targeted biopsies, median (IQR)	0 (0–1)	3 (2–4)	**<0.001**
Systematic biopsies, median (IQR)	12 (12–12)	12 (12–12)	0.28
Positive systematic biopsies, median (IQR)	0 (0–1)	3 (1–5)	**<0.001**

*Note*: Bold text denotes statistical significance (*p* < 0.05).

### 3.2 PSAD and cancer detection

Table 2 summarizes PCa detection across PSAD intervals. Detection of GG ≥ 2 disease increased stepwise with PSAD, with absolute increases of 18.5% and 17.2% beyond the thresholds of 0.15 and 0.2 ng/ml/cc, respectively. A similar pattern was observed for GG ≥ 3 detection, with the greatest incremental gain (19.9%) occurring also at PSAD ≥0.2 ng/ml/cc. In contrast, among men with low PSAD (<0.1 ng/ml/cc), the GG ≥ 2 detection rate on combined biopsy was only 29.2%, and the likelihood of GG ≥ 3 disease was even lower at 11.8%. On multivariable analysis, log‐transformed PSAD was independently associated with detection of GG ≥ 2 (OR 2.4, 95% CI 1.73–3.3, *p* < 0.001) and GG ≥ 3 (OR 2.3, 95% CI 1.65–3.2, *p* < 0.001) cancer, indicating a greater than twofold increase in risk with each doubling of PSAD (Table 3).

**TABLE 2:** Absolute cancer detection rate, stratified by PSA density.

	Cancer detection	
PSA density, ng/ml/cc	GG ≥ 2	GG ≥ 3
<0.1 (*N* = 144)	29.20% (22.2–36.9%)	11.80% (7.3–17.8%)
0.1–0.1499 (*N* = 89)	42.70% (32.8–53.1%)	21.30% (13.8–30.7%)
0.15–0.1999 (*N* = 49)	61.20% (47.3–73.9%)	32.70% (20.8–46.5%)
≥0.2 (*N* = 97)	78.40% (69.4–85.6%)	52.60% (42.7–62.3%)

**TABLE 3:** Multivariable logistic regression models for detection of GG ≥ 2 and GG ≥ 3 prostate cancer.

Variable	GG ≥ 2		GG ≥ 3	
	OR (95% CI)	*p* value	OR (95% CI)	*p* value
Age	1.08 (1.04–1.12)	**<0.001**	1.05 (1.02–1.09)	**0.005**
Race				
White (ref)	–		–	
Non‐White	0.51 (0.3–0.86)	**0.012**	0.49 (0.27–0.89)	**0.019**
Family history of prostate cancer (brother or father)	1.49 (0.82–2.7)	0.19	0.83 (0.41–1.68)	0.6
Biopsy history				
Biopsy‐naïve (ref)	–	–	–	–
Prior negative	0.17 (0.067–0.44)	**<0.001**	0.38 (0.14–1.03)	0.058
PI‐RADS score				
PI‐RADS 3 (ref)	–	–	–	–
PI‐RADS 4	2.04 (1.04–4)	**0.037**	3.76 (1.24–11.4)	**0.019**
PI‐RADS 5	5.68 (2.61–12.4)	**<0.001**	10.3 (3.32–31.9)	**<0.001**
log_2_ (prostate volume)	0.65 (0.431–0.975)	**0.037**	0.9 (0.577–1.406)	0.6
log_2_ (PSA density)	2.39 (1.73–3.3)	**<0.001**	2.3 (1.65–3.2)	**<0.001**

*Note*: Bold text denotes statistical significance (*p* < 0.05).

PSAD was also evaluated within PI‐RADS groups using a low cutoff of <0.1 ng/ml/cc, which is traditionally considered low‐risk (Table S1). In men with PI‐RADS 3 (*n* = 83) or PI‐RADS 4 (*n* = 185), the GG ≥ 2 detection rate approximately doubled when PSAD was at or above 0.1 ng/ml/cc (14.6% vs. 28.6% for PI‐RADS 3, 26.0% vs. 59.3% for PI‐RADS 4). For PI‐RADS 5, GG ≥ 2 detection increased from 61.5% to 80.0%, but GG ≥ 3 detection more than doubled from 26.9% to 62.4%.

### 3.3 PSAD and biopsy strategy

As PSAD increased, GG ≥ 2 and GG ≥ 3 cancer detection increased monotonically for both targeted and systematic biopsy when considered as standalone strategies. When PSAD exceeded 0.2 ng/ml/cc, there was an absolute increase in cancer detection by targeted biopsy of 24.2% and 26.0% for GG ≥ 2 and GG ≥ 3 disease. A more modest effect was seen for systematic biopsy, which detected an additional 12.9% and 10.6% of GG ≥ 2 and GG ≥ 3 cancer beyond this threshold. Notably, crossing this threshold was also associated with a marked reduction in GG 1 detection on targeted biopsy (26.5% to 9.3%), whereas GG 1 detection on systematic biopsy did not change (49.0% to 48.5%).

The absolute increase in cancer detection rate with the inclusion of targeted or systematic biopsy in addition to the alternative strategy was further stratified by PSAD (Table 4). For example, the systematic biopsy column represents the additional cancer detection associated with adding systematic biopsy to targeted biopsy. Conversely, the targeted biopsy column represents the additional cancer detection gained by adding targeted biopsy to systematic biopsy. Across PSAD intervals, adding systematic biopsy to targeted biopsy was associated with disproportionate added detection of GG 1 cancer compared to GG ≥ 2 or GG ≥ 3 cancer. This disparity was most apparent for PSAD ≥0.2 ng/ml/cc, where systematic cores detected an additional 42.3% GG 1, with an incremental GG ≥ 2 and GG ≥ 3 yield of only 7.2% and 6.2%. In practical terms, this implies that omitting systematic biopsy in this population risks missing clinically significant cancer in 1 in approximately 14 men while avoiding detection of indolent disease in 1 in less than 3 men. In comparison, for PSAD between 0.15 and 0.1999 ng/ml/cc, the tradeoff between GG 1 versus GG ≥ 2 or GG ≥ 3 detection was 30.6% vs. 14.3% or 12.2%.

**TABLE 4:** Absolute contribution to cancer detection rate by targeted or systematic biopsy, when added to the alternative strategy, stratified by PSA density.

	GG 1		GG ≥ 2		GG ≥ 3	
PSA density, ng/ml/cc	TB	SB	TB	SB	TB	SB
Overall (*N* = 379)	6.6% (4.5–9.2%)	26.4% (21.6–31.1%)	13.4% (10.0–16.9%)	6.9% (4.8–9.8%)	6.3% (4.0–8.7%)	5.5% (3.4–7.9%)
<0.1 (*N* = 144)	8.3% (4.2–12.5%)	15.3% (9.7–21.5%)	10.4% (6.3–15.3%)	3.5% (0.7–6.9%)	2.8% (0.7–5.6%)	2.1% (0–4.9%)
0.1–0.1499 (*N* = 89)	6.7% (2.3–12.3%)	24.7% (16.9–33.7%)	12.4% (5.6–19.1%)	7.9% (2.3–13.5%)	2.3% (0–5.6%)	6.7% (2.3–12.4%)
0.15–0.1999 (*N* = 49)	8.2% (2.0–16.3%)	30.6% (18.4–44.9%)	14.3% (6.1–24.5%)	14.3% (6.1–24.5%)	6.1% (0–12.2%)	12.2% (4.1–22.5%)
≥0.2 (*N* = 97)	3.1% (0–7.2%)	42.3% (33.0–51.6%)	18.6% (11.3–26.8%)	7.2% (2.1–12.4%)	15.5% (8.3–22.7%)	6.2% (2.1–11.3%)

### 3.4 Pathologic concordance

**FIGURE 1:**
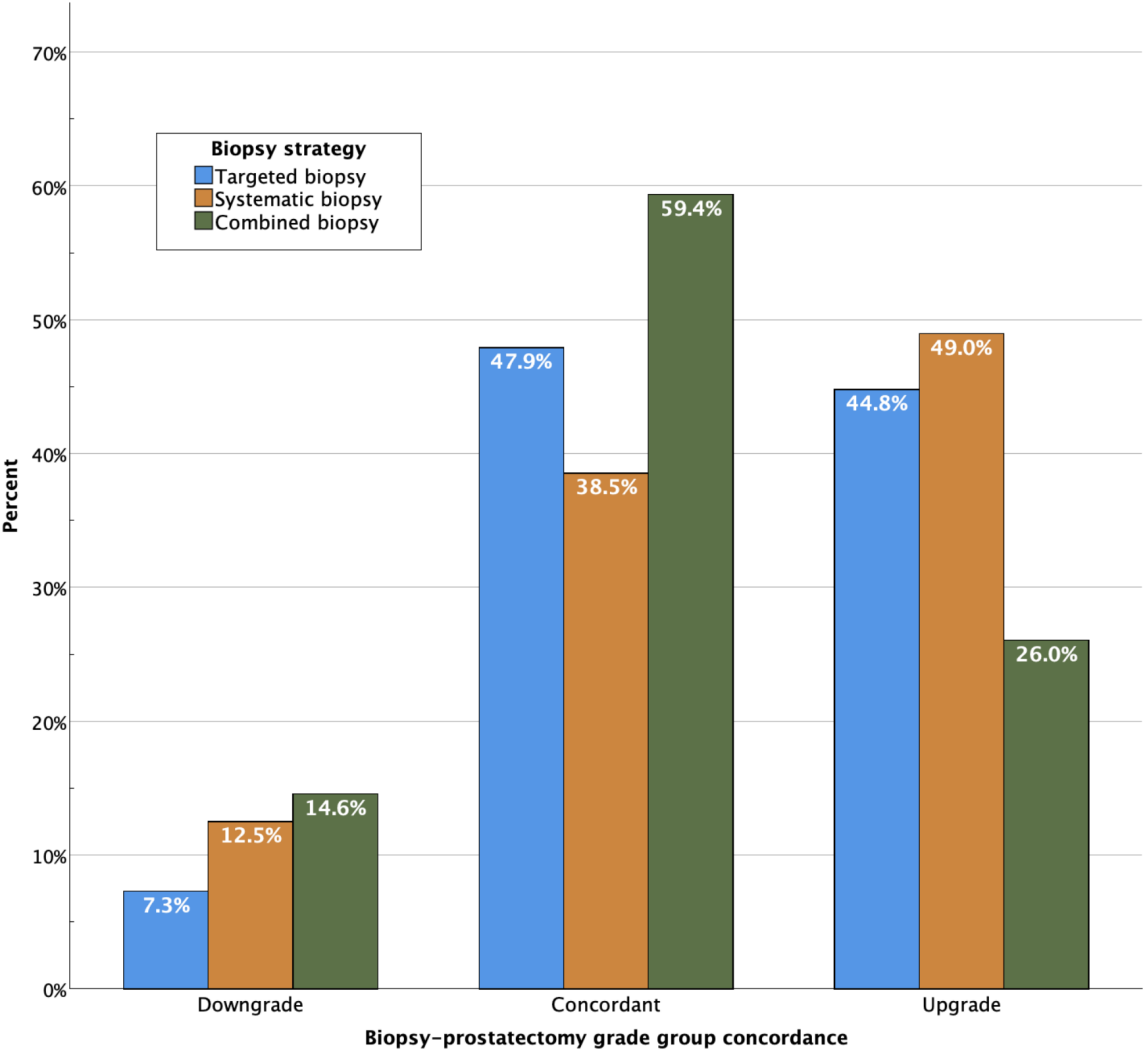
Grade group concordance between biopsy strategies and radical prostatectomy specimens.

Among 96 (25.3%) patients who proceeded to radical prostatectomy, combined biopsy demonstrated the strongest agreement with grade group at prostatectomy (κ = 0.747, *p* < 0.001), compared with targeted biopsy alone (κ = 0.575, *p* < 0.001) and systematic biopsy alone (κ = 0.443, *p* < 0.001) (Figure 1). In those with PSAD ≥ 0.2 ng/ml/cc (*n* = 31/96, 32.3%), the difference in agreement between targeted and combined biopsy was attenuated (κ = 0.584 vs. 0.628), suggesting a more limited contribution of systematic cores to overall pathologic concordance within this group.

## 4 DISCUSSION

In this study, we evaluated PSAD as a predictive marker for csPCa and as a tool for selection of biopsy template in men undergoing MRI‐guided transperineal biopsy. A recent secondary analysis of the Trio study showed that adding systematic biopsy led to a 2% incremental GG ≥ 2 yield when PSAD exceeded 0.2.^14^ Whereas the existing evidence is derived from transrectal biopsy, we sought to validate this cutoff for transperineal biopsy. We found that systematic biopsy was associated with a tradeoff of 7.2% added GG ≥ 2 detection versus 42.3% added GG 1 detection at PSAD ≥ 0.2, which meets our a priori criteria for omission. These findings support the notion that, in addition to its discriminative value, an elevated PSAD ≥ 0.2 may identify patients in whom omission of systematic sampling can be considered.

On multivariable analysis, log‐transformed PSAD was independently associated with GG ≥ 2 and GG ≥ 3 disease, with each doubling of PSAD conferring a more than twofold increase in risk. Although PSAD was originally developed in the era of ultrasound‐guided transrectal biopsy, it remains a robust predictor in the modern era. Though frequently dichotomized at 0.15, the optimal PSAD cutoff may vary across different populations. For example, Durmaz et al.^18^ identified 0.2 as the optimal cutoff for PI‐RADS 1–2 lesions and 0.12 for PI‐RADS 3 lesions. Yang et al.^19^ further demonstrated that PI‐RADS lesions in the peripheral zone were associated with greater PSAD and therefore should trigger biopsy at higher PSAD thresholds relative to transition zone tumours.

We posit that PSAD may be applied to help clinicians optimize prostate biopsy and increase procedural efficiency. Risk‐adapted selection of targeted versus systematic biopsy can reduce overdiagnosis of indolent disease, thereby limiting unnecessary surveillance and potential downstream overtreatment.^20^, ^21^ Fewer biopsy cores would also decrease patient discomfort, pathology workload and the risk of infectious and urinary complications.^6^, ^22^, ^23^, ^24^

A growing body of evidence has supported prioritization of targeted cores and de‐escalation of systematic cores in the setting of transrectal biopsy. In the GÖTEBORG‐2 trial, only 2.9% of patients undergoing combined biopsy had GG ≥ 2 PCa detected exclusively by systematic sampling.^25^ Patel et al.^26^ showed in a large multi‐institutional cohort that the addition of targeted biopsy to systematic biopsy increased GG ≥ 2 detection by 10.0% while reducing GG 1 detection by 0.5%, but, conversely, the addition of systematic biopsy to targeted biopsy added more GG 1 detection (6.0%) than GG ≥ 2 (5.4%). Elsewhere, studies have shown that omitting systematic cores in those with a prior negative systematic biopsy or PI‐RADS 5 lesions would miss only 1.3% and 1% of GG ≥ 2 PCa, respectively.^13^, ^27^ Furthermore, the concept of a cancer ‘penumbra’ that surrounds MRI‐visible lesions has also driven interest in regional strategies, such as side‐specific biopsy or perilesional biopsy, in place of whole‐gland systematic sampling.^28^

Though the current data are promising, systematic biopsy remains useful in some contexts. Given the limitations of MRI, systematic biopsy can identify MRI‐occult lesions, which, although often associated with more favourable biology and prognosis, may still impact management.^29^, ^30^ Systematic cores also provide more comprehensive disease characterization, as evidenced by improved pathologic concordance on combined biopsy relative to targeted biopsy. This information may help guide surgical planning prior to radical prostatectomy and patient selection for focal therapy.

A few limitations should be considered. First, the retrospective, single‐centre design of this study may limit generalizability, given our institution is a tertiary academic centre with potential referral and case‐mix bias. In addition, inclusion of MRI studies performed at outside facilities introduces variability in image acquisition and interpretation, though it reflects real‐world practice. Heterogeneity in prostate segmentation and ROI annotation among different operators may also affect targeting accuracy and estimates of diagnostic yield. A key strength is the large transperineal biopsy cohort, which increasingly reflects contemporary and future practice.

## 5 CONCLUSIONS

PSAD is an easily accessible tool for biopsy risk stratification. Although GG ≥ 2 cancer detection increases with PSAD, systematic sampling at PSAD ≥ 0.2 added limited yield of clinically significant disease relative to low‐grade disease. Incorporating PSAD thresholds may help guide selective omission of systematic sampling in appropriately counselled patients.

## AUTHOR CONTRIBUTIONS

**Johnny Wang**: Conceptualization; methodology; investigation; data curation; formal analysis; writing—original draft; writing—review and editing. **Narmina Khanmammadova**: Conceptualization; methodology; data curation. **Karim Hanna**: Investigation; writing—review and editing. **Ashley Gao**: Investigation; data curation. **Angelina Wang**: Investigation; data curation; writing—review and editing. **Kimberly Tran**: Investigation; data curation. Mohamad Ibrahim: Investigation; data curation. **Mackinnly Knoerzer**: Investigation; data curation; writing—review and editing. **Kristene Myklak**: Investigation; data curation; writing—review and editing. **Sohrab Ali**: Investigation; data curation; writing—review and editing. **Michael Daneshvar**: Investigation; data curation; writing—review and editing; supervision. **Mohammed Shahait**: Conceptualization; methodology; formal analysis; writing—review and editing; supervision. **David Lee**: Conceptualization; methodology; writing—review and editing; supervision.

## CONFLICT OF INTEREST STATEMENT

The authors have no competing interests to declare that are relevant to the content of this article.

## Supporting information


**Figure S1.** Institutional transperineal biopsy template.

**Table S1.** Cancer detection rates within PI‐RADS and PSA density subgroups.

## Data Availability

The data that support the findings of this study are not publicly available due to potentially identifiable patient information, but de‐identified data may be made available from the corresponding author on reasonable request and with institutional approval.
