# Supplementary figures and images for: The utility of PSA density for selection of targeted versus systematic transperineal prostate biopsy: A retrospective cohort study
Source: BJUI Compass. 2026 Jul 14;7(7):e70247. doi: 10.1002/bco2.70247 (PMC13369285; doi:10.1002/bco2.70247)

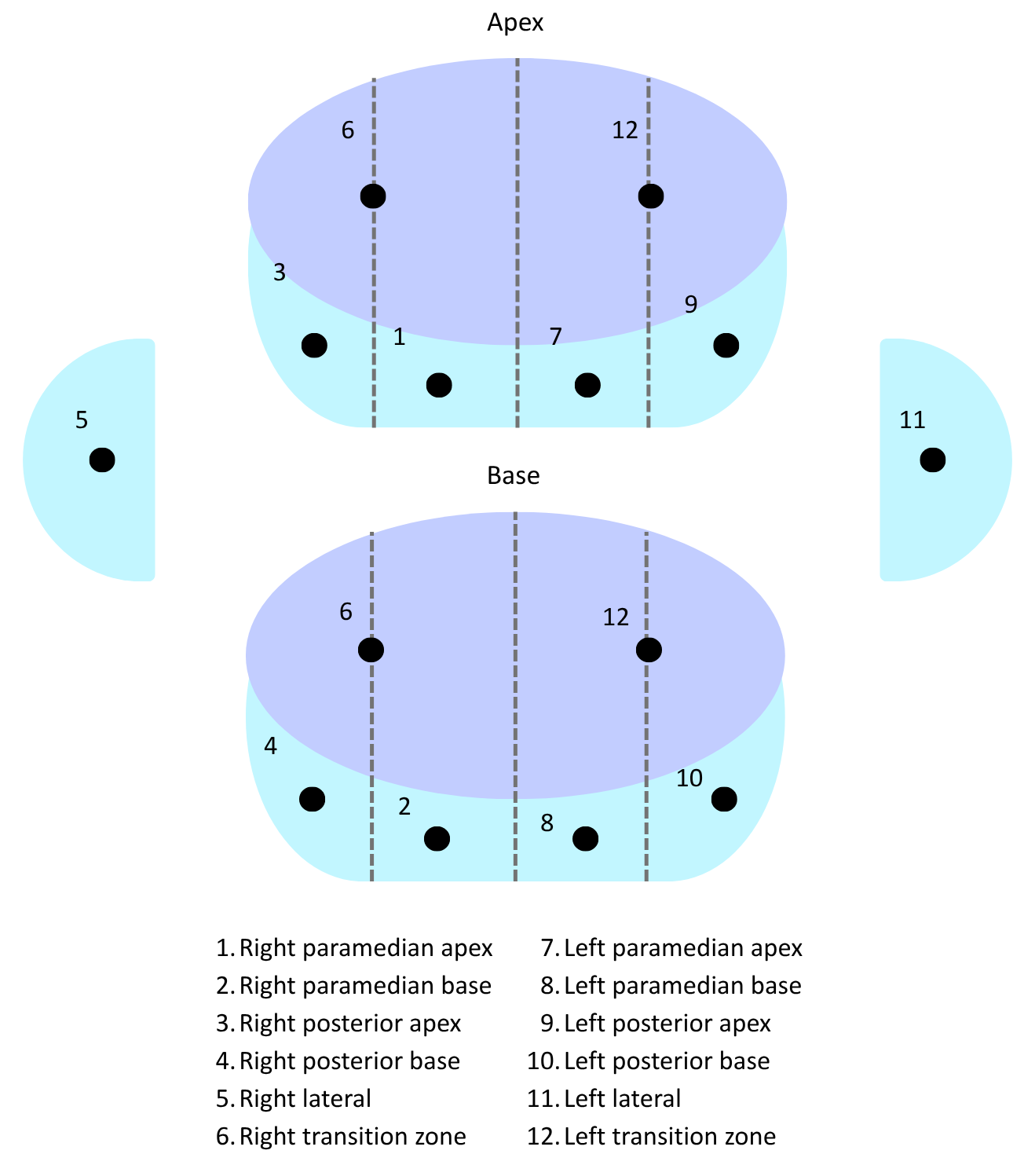

Supplement: Supplementary file 1 — Figure S1. Institutional transperineal biopsy template. [file BCO2-7-e70247-s002.png]
