# Supplementary material for: The utility of PSA density for selection of targeted versus systematic transperineal prostate biopsy: A retrospective cohort study
Source: BJUI Compass. 2026 Jul 14;7(7):e70247. doi: 10.1002/bco2.70247 (PMC13369285; doi:10.1002/bco2.70247)
Supplement: Supplementary file 2 — Table S1. Cancer detection rates within PI‐RADS and PSA density subgroups. [file BCO2-7-e70247-s001.docx]

Supplementary Table 1. Cancer detection rates within PI-RADS and PSA density subgroups

| Index PIRADS | n | PSA density | GG ≥2 | GG ≥3 |
| --- | --- | --- | --- | --- |
| 3 | 41 | <0.10 | 14.6% (6.3%-27.7%) | 0% |
|  | 42 | ≥0.10 | 28.6% (16.7%-43.3%) | 9.5% (3.3%-21.1%) |
| 4 | 77 | <0.10 | 26.0% (17.2%-36.5%) | 13.0% (6.9%-21.8%) |
|  | 108 | ≥0.10 | 59.3% (49.8%-68.2%) | 26.9% (19.2%-35.7%) |
| 5 | 26 | <0.10 | 61.5% (42.4%-78.2%) | 26.9% (12.9%-45.7%) |
|  | 85 | ≥0.10 | 80.0% (70.6%-87.4%) | 62.4% (51.8%-72.1%) |
